# Iatrogenic Aortic Insufficiency Following Mitral Valve Replacement: Case Report and Review of the Literature

**DOI:** 10.14740/jocmr2128w

**Published:** 2015-04-08

**Authors:** Pavani Kolakalapudi, Sadaf Chaudhry, Bassam Omar

**Affiliations:** a Division of Cardiology, University of South Alabama, Mobile, AL 36617, USA

**Keywords:** Aortic valve, Mitral valve, Endocarditis, Surgical complication

## Abstract

We report a 28-year-old white female who suffered significant aortic insufficiency (AI) following mitral valve (MV) replacement for endocarditis. The patient had history of rheumatoid arthritis and presented to our emergency department with a 3-month history of dyspnea, orthopnea, fevers and weight loss, worsening over 2 weeks, for which she took intermittent acetaminophen. On admission, vital signs revealed blood pressure of 99/70 mm Hg, heart rate of 120 beats/minute, and temperature of 98.8 °F; her weight was 100 lbs. Physical exam revealed a thin and pale female. Cardiac auscultation revealed regular tachycardic rhythm with a third heart sound, and a short early systolic murmur at the left lower sternal border without radiation. Lungs revealed right lower lobe rhonchi. Initial pertinent laboratory evaluation revealed hemoglobin 9.6 g/dL and white blood cell count 17,500/μL. Renal function was normal, and hepatic enzymes were mildly elevated. Chest radiogram revealed right lower lobe infiltrate. Blood cultures revealed *Enterococcus faecalis*. Two-dimensional echocardiogram revealed large multilobed vegetation attached to the anterior MV leaflet with severe mitral regurgitation (MR), otherwise normal left ventricular systolic function. She was started on appropriate antibiotics and underwent MV replacement with 25-mm On-X prosthesis. She was noted post-operatively to have prominent systolic and diastolic murmurs. Repeat echocardiogram revealed normal mitral prosthesis function, with new moderately severe AI. Transesophageal echocardiogram revealed AI originating from a tethered non-coronary cusp, due to a suture preventing proper cusp mobility. The patient declined further surgery. She recovered slowly and was discharged to inpatient rehabilitation 4 weeks later. This case highlights the importance of vigilance to this potential serious complication of valve surgery with regard to diagnosis and treatment to prevent long-term adverse consequences.

## Introduction

Iatrogenic valvular insufficiency has been reported following different cardiac procedures as a result of leaflet entrapment or perforation. The aortic valve (AV) appears to be more vulnerable to such potential complication given its central location and proximity of the individual cusps to various cardiac structures. The specific AV cusp involved appears to depend on the proximity of the surgery performed to that particular cusp.

## Case Report

**Figure 1:**
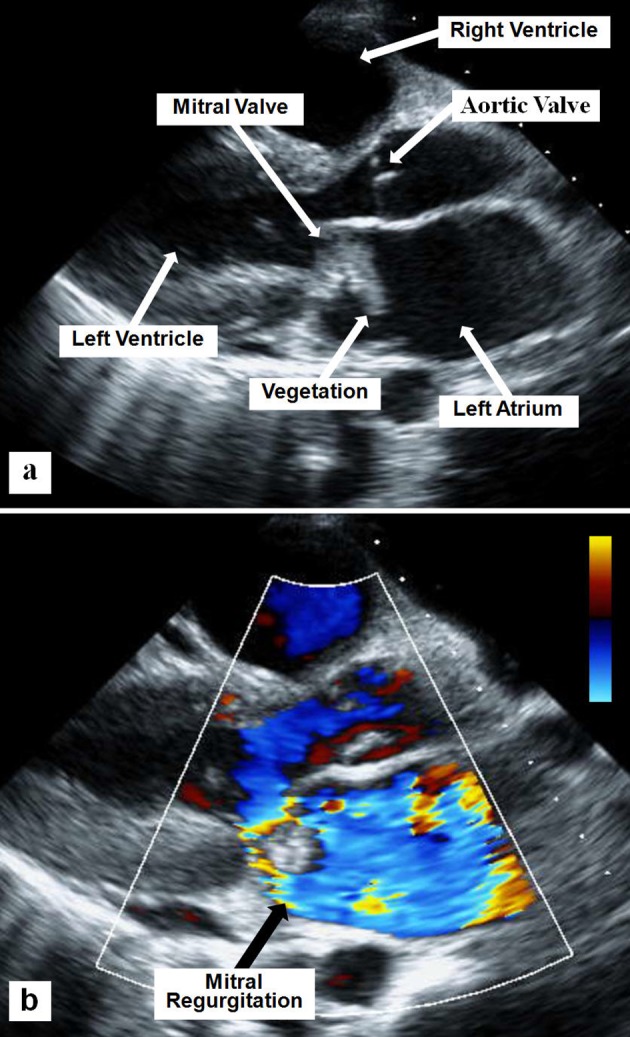
Two-D parasternal long axis echocardiographic view showing the mitral valve vegetation (a) and the severe mitral regurgitation using superimposed color flow Doppler (b).

**Figure 2:**
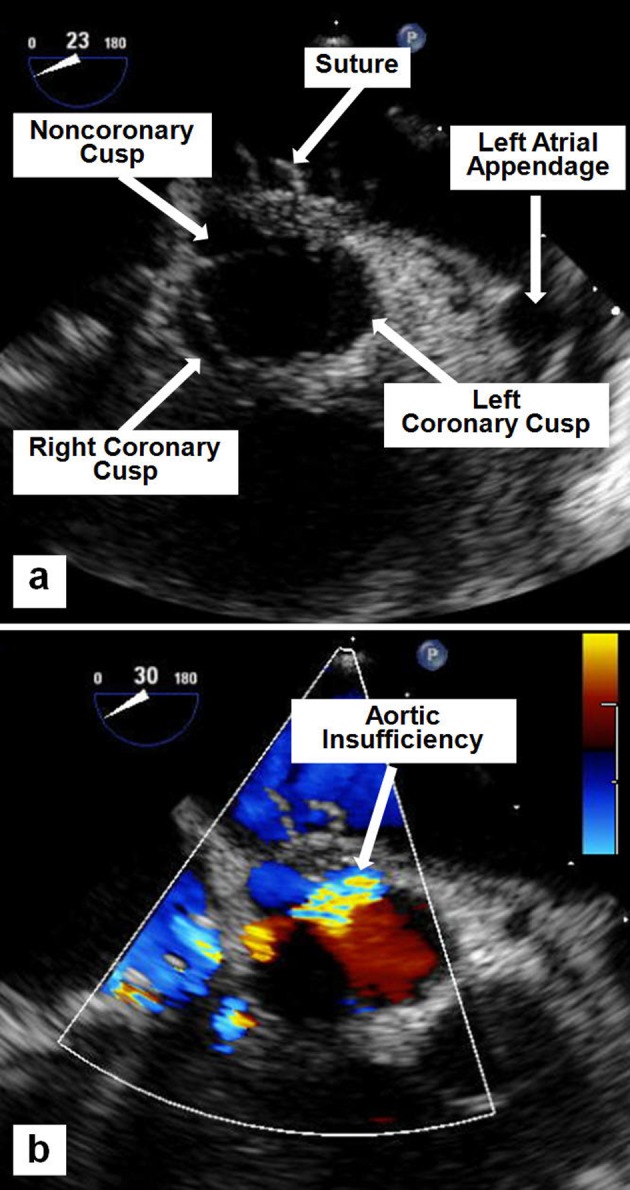
Mid-esophageal TEE images showing a suture tethering the non-coronary cusp (a), and the resulting moderate-to-severe AI by color flow Doppler (b).

A 28-year-old white female with history of rheumatoid arthritis presented to our emergency department with a 3-month history of dyspnea, orthopnea, fevers and weight loss, worsening over 2 weeks, for which she took intermittent acetaminophen. On admission, vital signs revealed blood pressure of 99/70 mm Hg, heart rate of 120 beats/min, and temperature of 98.8 °F; her weight was 100 lbs. Physical exam revealed a thin and pale female. Cardiac auscultation revealed regular tachycardic rhythm with a third heart sound, and a short early systolic murmur at the left lower sternal border without radiation. Lungs revealed right lower lobe rhonchi. Initial pertinent laboratory evaluation revealed hemoglobin 9.6 g/dL and white blood cell count 17,500/μL. Renal function was normal, and hepatic enzymes were mildly elevated. Chest radiogram revealed right lower lobe infiltrate. Blood cultures revealed *Enterococcus faecalis*. Two-dimensional echocardiogram revealed large multilobed vegetation attached to the anterior mitral valve (MV) leaflet with severe mitral regurgitation (MR) (Fig. 1), otherwise normal left ventricular systolic function. She was started on appropriate antibiotics and underwent MV replacement with 25-mm On-X prosthesis. She was noted post-operatively to have prominent systolic and diastolic murmurs. Repeat echocardiogram revealed normal mitral prosthesis function, with new moderately severe aortic insufficiency (AI). Transesophageal echocardiogram revealed AI originating from a tethered non-coronary cusp, due to a suture preventing proper cusp mobility (Fig. 2). The patient declined further surgery. She recovered slowly and was discharged to inpatient rehabilitation 4 weeks later.

## Discussion

Iatrogenic valvular insufficiency due to leaflet damage can affect cardiac valves following surgery in their vicinity. The AV appears to be particularly vulnerable to damage by various cardiac surgical procedures due to its central location. Diagnosis and treatment of iatrogenic leaflet perforation depends on the location and severity of valvular insufficiency, with wide variations and challenges reported in the literature, as will be discussed next.

Hill et al [1] reported six cases of iatrogenic AI following non-aortic valve operations. Five of these cases required reoperation; two underwent AV repair and two had AV replacement, while the fifth case required heart transplantation. The sixth patient died of transfusion reaction before any reoperation. The location of the AV cusp perforation varied and depended on the site of the original intracardiac lesion repair (Table 1) [1-13].

**Table 1:** Summary of Reports Demonstrating Relationship of Procedure Performed to Aortic Coronary Cusp Involved

	Non-coronary cusp	Right coronary cusp	Left coronary cusp
Hill et al [1]	MVR (two cases)	VSD repair	MVR
	ASD repair		
	VSD repair		
Ducharme et al [2]			MVA
Rother et al [3]			MVA
Aboelnasr and Rohn [4]	MVA		
Mehta and Hunsaker [5]			MVA
Santiago et al [6]	MVR		MVA
Dogan et al [7]	MVR		
Al Yamani et al [8]		MVR	
Oakley et al [9]	MVR		
Dreyfus et al [10]	MVA		
Pagel et al [11]	MVA		
Rey et al [12]	ASD repair (nine cases)		
Woo et al [13]		VSD repair	

MVA: mitral valve annuloplasty ring; MVR: mitral valve replacement.

Ducharme et al [2] reported a case of severe AI after insertion of a flexible Carpentier ring during MV repair in a 54-year-old man with moderate to severe MR undergoing bypass surgery for left main disease. Severe AI was diagnosed intraoperatively by transesophageal echocardiography (TEE) originating from the left coronary cusp, which was corrected by releasing a few sutures on the annuloplasty ring, with a subsequently uneventful postoperative recovery.

Rother et al [3] reported a 46-year-old man who, after elective MV repair for severe MR using a Carpentier-Edwards annuloplasty ring, was found to have a non-mobile left coronary cusp of the AV on intraoperative TEE, causing moderate AI. Surgical exploration of the aortic root revealed that an MV annuloplasty ring suture had perforated and tethered the left coronary cusp. Despite release of the tethering suture, the damaged AV leaflet was surgically unrepairable, necessitating AV replacement.

Aboelnasr and Rohn [4] reported a 32-year-old man with AV non-coronary cusp perforation resulting in severe AI after MV repair using a Carpentier-Edwards annuloplasty ring, which was repaired with pericardial patch. The postoperative course was reported to be uneventful.

Mehta and Hunsaker [5] reported severe AI due to a suture inadvertently placed in the left coronary cusp in a 79-year-old woman who underwent MV repair using Carpentier-Edwards annuloplasty ring insertion for severe MR. She underwent AV replacement with a porcine prosthesis; however, she died of excess intraoperative bleeding.

Santiago et al [6] reported two cases of iatrogenic AI. The first case was detected 6 days following coronary artery bypass grafting (CABG) and MV St. Judes saddle ring repair on a 37-year-old woman with cardiogenic shock and severe MR. The left coronary cusp was found to have restricted motion resulting in severe AI on a follow-up TEE performed because of decreased activity tolerance; the patient was successfully treated with an AV bioprosthesis, as the valve was not amenable to repair. The second case was an intraoperatively detected suture in the non-coronary cusp resulting in severe AI following replacement of a dysfunctional mechanical mitral prosthesis with a Carpentier-Edwards pericardial valve in a 67-year-old woman. The patient received an AV bioprosthesis, and recovered slowly after sustaining a hemorrhagic stroke.

Dogan et al [7] reported two cases of iatrogenic valvular perforation following valve replacement surgery involving the aortic and MVs. One case was MV replacement on a 30-year-old man due to rheumatic MR 10 years earlier, who was found to have perforation of the non-coronary cusp of his AV resulting in mild AI, in the absence of any evidence of endocarditis; he was followed medically. The second was a unique case of mitral perforation along the mitral-aortic intervalvular fibrosa region on the anterior MV leaflet resulting in significant MR, detected several years following reoperation on the AV in a 50-year-old woman. She was also followed up with medical therapy, as she refused surgery.

Al Yamani et al [8] reported a rare right coronary cusp perforation following MV replacement in a 60-year-old man for severe symptomatic MR. The acute severe AI was treated with a supra-annular aortic mechanical prosthesis; the patient, however, died of multiorgan failure.

TEE is usually the modality of choice for detecting valvular perforation [14]. Traditional TEE views, however, may not be adequate, especially if surgical sutures impede the mobility of a mechanical aortic prosthesis disks. Koh and Gandhi [15] reported a case of mechanical tilting disk aortic prosthesis malfunction following MV replacement for rheumatic mitral stenosis in a 50-year-old man. The traditional mid-esophageal TEE views were not helpful due to excess shadowing over the aortic prosthesis from the newly placed MV prosthesis. Deep transgastric views, however, revealed severe eccentric AI caused by the sutures used to secure the mitral prosthesis, which necessitated repositioning of the AV prosthesis. Oakley et al [9], on the other hand, demonstrated that while TEE failed to show iatrogenic AI due to perforation of the non-coronary cusp following repeat MV replacement in a 24-year-old man with prosthetic MV endocarditis, cardiac gated computed tomography (CT) was able to fully characterize the defect.

The advent of three-dimensional (3D) TEE provided enhanced imaging of valves, with the added benefit of diagnosing valvular perforation previously not identifiable by two-dimensional echocardiography [16]. Dreyfus et al [10] demonstrated, using 3D TEE, iatrogenic perforation of the non-coronary cusp of the AV resulting in mild AI in a 48-year-old man 5 years after CABG and MV annuloplasty for ischemic MR, in the absence of any evidence of AV endocarditis. Van Gorselen et al [17] used 3D TEE to demonstrate a rare left ventricular outflow tract to left atrial severe regurgitation due to perforation of the mitral-aortic intervalvular fibrosa following AV replacement in a 91-year-old man; he was not a candidate for percutaneous therapy and was therefore treated medically.

Repair of other congenital heart lesions in close proximity to the AV may also place the aortic valve at risk of cusp tethering or perforation. Pagel et al [11] reported a case of endocardial cushion defect undergoing mitral and tricuspid valve repair, resulting in iatrogenic AI due to tethering of the non-coronary cusp by mitral annuloplasty sutures. Rey et al [12] reported eight children with repaired ostium primum atrial septal defects (ASDs) causing perforation of the non-coronary cusp of the AV and resulting in varying degrees of AI, some requiring surgical intervention. Woo et al [13] reported a 22-year-old woman with mild to moderate AI due to AV right coronary cusp perforation detected 15 years following simple membraneous ventricular septal defect (VSD) repair, in the absence of any evidence of endocarditis. Zhang et al [18] reported a similar case of iatrogenic AI following simple VSD closure, which required repair using a pericardial patch. Sabzi et al [19] reported a 15-year-old boy who underwent VSD repair with a Dacron patch, combined with subvalvular pulmonic stenosis repair with RVOT muscle resection and pericardial patch. He developed significant AI due to tearing of the non-coronary cusp, presumably due to the separation of the cusp from its ring caused by tension created by the Dacron patch pulling on the adjacent tissue. Left ventricular septal myectomy has been shown to result in new AI requiring repair in 5.5% of patients, and new MR requiring repair in 1.5% of patients [20].

### Conclusion

**Figure 3:**
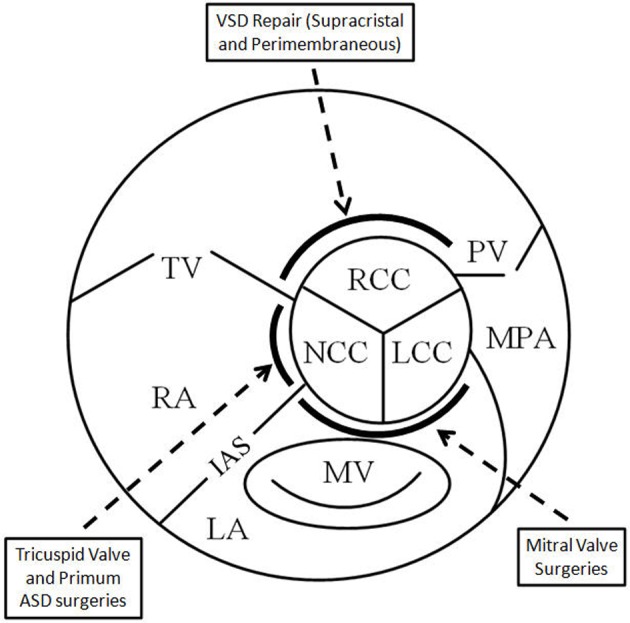
Schematic showing the central location of the aortic valve and the vulnerability of the cusps in relation to adjacent surgery. RCC: right coronary cusp; LCC: left coronary cusp; NCC: non-coronary cusp; TV: tricuspid valve; MV: mitral valve; PV: pulmonic valve; MPA: main pulmonary artery; LA: left atrium; RA: right atrium; IAS: interatrial septum.

Damage to AV cusps resulting in varying degrees of insufficiency has been reported following various surgeries in the vicinity of the centrally located AV. Different modalities and techniques have been employed to identify such damage. The location of the damage usually is a factor of the specific non-aortic valve surgery being performed, and the close proximity of sutures to a particular AV cusp (Table 1, Fig. 3). Vigilance is recommended with regard to such potentially life-threatening complications, employing proper, often multimodality, imaging techniques for diagnosis so that early intervention may be implemented.
